# Expression Profiles and DNA-Binding Affinity of Five ERF Genes in Bunches of *Vitis vinifera* cv. Cardinal Treated with High Levels of CO_2_ at Low Temperature

**DOI:** 10.3389/fpls.2016.01748

**Published:** 2016-11-28

**Authors:** Irene Romero, Maria Vazquez-Hernandez, M. I. Escribano, Carmen Merodio, M. T. Sanchez-Ballesta

**Affiliations:** Departamento de Caracterización, Calidad y Seguridad, Instituto de Ciencia y Tecnología de Alimentos y Nutrición, Consejo Superior de Investigaciones Científicas, Ciudad UniversitariaMadrid, Spain

**Keywords:** ERF, transcription factors, *Vitis vinifera*, carbon dioxide, low temperature

## Abstract

Ethylene response factors (ERFs) play an important role in plants by regulating defense response through interaction with various stress pathways. After harvest, table grapes (*Vitis vinifera* L.) are subject to a range of problems associated with postharvest storage at 0°C, such as fungal attack, water loss and rachis browning. The application of a 3-day high CO_2_ treatment maintained fruit quality and activated the induction of transcription factors belonging to different families such as ERF. In this paper, we have isolated five *VviERFs* from table grapes cv. Cardinal, whose deduced amino acid sequence contained the conserved apetalous (AP2)/ERF domain. The phylogeny and putative conserved motifs in VviERFs were analyzed and compared with those previously reported in *Vitis*. *VviERFs-c* gene expression was studied by quantitative real-time RT-PCR in the different tissues of bunches stored at low temperature and treated with high levels of CO_2_. The results showed that in most of the tissues analyzed, *VviERFs-c* gene expression was induced by the storage under normal atmosphere although the application of high levels of CO_2_ caused a greater increase in the *VviERFs-c* transcript accumulation. The promoter regions of two PRs (pathogenesis related proteins), *Vcchit1b* and *Vcgns1*, were obtained and the *in silico* analysis revealed the presence of a *cis*-acting ethylene response element (GCC box). In addition, expression of these two PR genes was analyzed in the pulp and rachis of CO_2_-treated and non-treated table grapes stored at 0°C and results showed significant correlations with *VviERF2-c* and *VviERF6L7-c* gene expression in rachis, and between *VviERF11-c* and *Vcchit1b* in pulp. Finally by using electro mobility shift assays, we denoted differences in binding of VviERFs to the GCC sequences present in the promoters of both PRs, with VviERF6L7-c being the only member which did not bind to any tested probe. Overall, our results suggest that the beneficial effect of high CO_2_ treatment maintaining table grape quality seems to be mediated by the regulation of *ERFs* and in particular *VviERF2-c* might play an important role by modulating the expression of PR genes.

## Introduction

The APETALA2/ethylene response factor (AP2/ERF) family is a large group of plant-specific transcription factors which includes four major sub-families, namely AP2, DREB (dehydration responsive element binding), ERF and RAV. The subdivision of the AP2/ERF transcription factors into the different families is based on the number of AP2 domains present in the proteins. Thus, ERF proteins contain a single highly conserved AP2/ERF domain consisting of 58–59 amino acids which can bind to the GCC box, with the core AGCCGCC sequence, present in the promoter of pathogenesis-related (PR) genes (Ohme-Takagi and Shinshi, 1995), and to the dehydration-responsive element (DRE)/CRT element, with the core A/GCCGAC sequence, found in the promoter region of stress responsive genes (Eini et al., 2013). The transcription of *ERFs* can be regulated in the frame of development and growth programs (reviewed by Licausi et al., 2013). Likewise, different studies have reported that ERF genes play a part in the environmental stress responses in many plant species, especially in *Arabidopsis* (Park et al., 2011; Yang et al., 2011). In the case of wheat, overexpression of an ERF transcription factor, *TaPIE1*, positively regulated the defense responses to *Rhizoctonia cerealis* and freezing stress by activating defense- and stress-related genes downstream of the ethylene signaling pathway (Zhu et al., 2014). However, introducing antisense *Sl-ERF.B.3* in tomato plants reduced cell injury and increased tolerance to low temperature stress (Klay et al., 2014).

In the last few years, a great deal of interest has been shown in the study of *ERFs* in fruit given that they have to face environmental stress during development and postharvest storage. In this sense, the storage of papaya fruit at 7°C induced gene expression of four *ERFs* (Li et al., 2013). In grapefruit, *ERF2* seems to be involved in the cascade of events induced during cold stress and that are negatively regulated by ethylene, since the application of the inhibitor of ethylene perception 1-methylcyclopropene (1-MCP) overstimulated their expression (Lado et al., 2015). Different works showed that this response was not only confined to low temperature stress. Thereby, Severo et al. (2015) established a relationship between Ultraviolet-C (UV-C) treatment and ripening delay in tomato fruit, correlated to changes in 13 *ERF* transcripts. The storage of apples at 1°C under hypoxic conditions (0.4 and 0.8 kPa oxygen) induced a higher expression of transcription factors including different *ERFs* (Cukrov et al., 2016). By other hand, the use of minimal processing operations during postharvest, such as wounding to obtain wedges in ripe peaches activated molecular responses including AP2/ERF transcription factors (Tosetti et al., 2014). Thus, the application of different postharvest treatments to maintain fruit quality seems to activate specific molecular changes affecting the transcriptional profiles of *ERFs*.

Grape (*Vitis vinifera* L.) is one of the most important fruit crops worldwide. As fresh fruit, table grapes are subject to serious water loss and fungal decay during postharvest handling at low temperature, which reduces their quality and limits their storage and marketing, leading to considerable economic losses. In previous studies, we have observed that applying 20 kPa CO_2_ for 3 days at 0°C reduced total decay and rachis browning in table grapes and retained their quality during postharvest (Romero et al., 2006; Sanchez-Ballesta et al., 2006; Rosales et al., 2016). Likewise, although table grapes have been classified as chilling-tolerant fruit, the CO_2_ pretreatment modified the cold- and antifungal-defense responses induced in non-treated table grapes, making them less noticeable (Romero et al., 2006; Sanchez-Ballesta et al., 2007). In a recent transcriptional analysis, we have shown that the maintenance of table grape quality by applying a 3-day high CO_2_ treatment seems to be an active process, requiring the activation of transcription factors belonging to different families such as ERF, as well as WRKY, MYB, basic-domain leucine-zipper (bZIP), heat stress transcription factor and zinc finger (Rosales et al., 2016). In addition, we have observed that the gaseous treatment induced the expression of *CBF1* and *CBF4* in the pulp as well as *CBF4* in the rachis of table grapes (Fernandez-Caballero et al., 2012). The fact that CBFs also belong to the AP2/ERF transcription factor family seems to indicate that this family could play a prominent role in the beneficial effect of the gaseous treatment in table grapes.

The grape genome sequence (Jaillon et al., 2007; Velasco et al., 2007) suggested that the *ERF* transcription factor family was composed of at least 122 members (Licausi et al., 2010a). The expression of the 122 *ERFs* from *Vitis* has been well characterized in aerial tissues such as stems, leaves and inflorescence, and during ripening in the skin and flesh of berries at veraison and full ripening (Licausi et al., 2010a). A transcriptome analysis of berry ripening under high temperatures showed a thermotolerance response coincident with the up-regulation of seven *ERFs* transcription factors (Carbonell-Bejerano et al., 2013). However, there is a lack of knowledge regarding the transcriptional regulation of *ERF* genes by different postharvest treatments in table grapes. In the present study, we isolated five ERFs from table grapes cv. Cardinal and investigated their gene expression in the different tissues of bunches (skin, pulp, seeds, and rachis) treated and non-treated with high levels of CO_2_ during postharvest storage at low temperature. Previously, we observed that expression levels of two PR genes, *Vcchit1b* and *Vcgns1*, increased in the skin of table grapes after 3-day storage at 0°C and the overexpression of both proteins in *Escherichia coli* showed *in vitro* cryoprotective activity and slightly antifungal activity (Romero et al., 2008; Fernandez-Caballero et al., 2009), indicating a putative protective role of these PRs in table grapes during storage at low temperature. In this work, we have isolated the promoter of *Vcchit1b* and *Vcgns1* and different *cis*-regulatory elements, including the GCC box, were identified. Furthermore, we have examined the DNA-binding specificities of the five ERFs, showing that four out of the five ERFs were able to bind *in vitro* to the GCC sequences presented in the *Vcchit1b* and *Vcgns1* promoters.

## Materials and Methods

### Plant Material

Table grapes (*V. vinifera* L. cv. Cardinal) were harvested from a commercial orchard located in Camas, Seville, Spain (latitude: 37°24′5″N; longitude: 6°2′4″W) in July 2003 at the early commercial stage (maturity index: 15.24). After harvesting at random, field-packaged bunches were transported in a refrigerated van in darkness to the laboratory in Madrid (Spain), where fruits were immediately forced-air precooled for 14 h at -1°C. Thereafter, bunches free from physical and pathological defects were randomly divided into two lots and stored at 0 ± 0.5°C and 95% relative humidity in two sealed neoprene containers of 1 m^3^ capacity. One lot was stored under normal atmosphere for up to 15 days (non-treated fruit) and the other under a gas mixture containing 20 kPa CO_2_ + 20 kPa O_2_ + 60 kPa N_2_ (CO_2_-treated fruit) for 3 days. After this time, CO_2_-treated grapes were transferred to air under the same conditions as non-treated fruits until the end of the storage period. At time 0 (after precooling at -1°C) and after 3 and 15 days of storage under air or CO_2_ conditions, berries from three biological replicates (each replicate consisting of two bunches) were peeled and the skin, pulp, seeds, and rachis were collected, frozen in liquid nitrogen, grounded to a fine powder, and stored at -80°C until analysis.

### RNA Extraction, cDNA Synthesis, and RT-PCR

Total RNA was extracted three times from each sample according to Zeng and Yang (2002), and treated with DNase I recombinant-RNase free (Roche) for genomic DNA removal. Then, 1 μg of each extraction was used to synthesize cDNA by using the iScript^TM^ Reverse Transcription Supermix (Bio-Rad). Three degenerate primers were used as first attempt to achieve different *ERFs* present in table grapes by PCR amplification. These primers were designed after the alignment of several ERFs from different plants which were obtained from Databases. Due to the fact that the ERF nucleotide alignment showed clearly two different consensus close to the 3′ end, two degenerated Reverse primers were synthetized to cover the variability observed (Supplementary Table S1). As template, a pool of cDNA synthesized using RNA extracted from the pulp and skin of CO_2_-treated and non-treated table grapes, was used. *VviERFs-c* full-lengths were obtained by RT-PCR as described by Romero et al. (2008) using specific primers (Supplementary Table S1). PCR fragments were cloned into pMBL-T vector (Canvax) and confirmed by Sanger sequencing at the Genomic Department of the Centro de Investigaciones Biológicas-Consejo Superior de Investigaciones Científicas (CIB-CSIC; Madrid, Spain). The five ERFs and the 122 known ERF proteins from *V. vinifera* (Licausi et al., 2010a) were used to run a search in the 12× grape reference genome, V2 gene prediction^1^ (Vitulo et al., 2014). The BLASTP and BLASTX suites were used to perform similarity searches in the predicted proteome database. Protein sequence identity between the closest ERFs homologs were performed by using the LALIGN program ^2^.

### Phylogenetic Tree

A phylogenetic tree was drawn using the 122 known ERF proteins from *V. vinifera* according to the name assigned in Licausi et al. (2010a) together with the VviERFs-c obtained in the present study. Sequences were aligned using ClustalW and the phylogenetic analyses were performed using MEGA software version 5.1. Minimum evolution and neighbor-joining trees were derived from the alignment with 1000 bootstrap replicates and default parameters.

### Relative Gene Expression by Quantitative Real-Time RT-PCR

Relative expression of *VviERFs-c*, class I β-1,3-glucanase (*Vcgns1*; GenBank accession no. DQ267748) and class I chitinase (*Vcchit1b*, GenBank accession no. DQ267094) were assayed using quantitative real-time RT-PCR (RT-qPCR) with samples of skin, pulp, seed, and rachis from CO_2_-treated and non-treated bunches stored for 0, 3, and 15 days at 0°C. RT-qPCR was performed as described by Rosales et al. (2013) using *Actin1* from *V. vinifera* (ACT1: XM 002282480) as the internal control. Gene-specific primers pairs for RT-qPCR (Supplementary Table S1) were designed with Primer 3 software (Koressaar and Remm, 2007). Each gene was evaluated at least in two independent runs. Relative expression levels were estimated by the 2-^ΔΔCt^ method, and denoted as the fold difference from the expression present at the calibrator sample (time 0). The specificity of products was validated by dissociation curve analysis and by agarose gel; and its sequences confirmed at the Genomic Department of the CIB-CSIC.

### Isolation of the *Vcchit1b* and *Vcgns1* Promoters

The 5′ upstream genomic sequences of *Vcchit1b* (GenBank accession no. KY048177) and *Vcgns1* (GenBank accession no. KY048178) were generated using a Universal GenomeWalker 2.0 Kit (Clontech) with nested PCR using genomic DNA from *V. vinifera* cv. Cardinal leaves isolated as described by Lodhi et al. (1994) with some modifications. Each genomic DNA aliquot was digested with four 6 bp-recognizing and blunt end-forming restriction enzymes *Dra*I, *Eco*RV, *Pvu*II, and *Stu*I. Adaptor DNA which harbored two primer-binding sites for AP1 and AP2 primers provided by the GenomeWalker Kit was linked to both ends of the restricted DNA fragment at 16°C. The nested PCR analysis was performed with the AP1 and AP2 primers obtained from the kit and two gene-specific antisense primers that were designed by Primer3, as described in Supplementary Table S1. The generated PCR product was cloned into pMBL-T vector (Canvax) and fully sequenced at the Genomic Department of the CIB-CSIC. Conserved *cis*-element motifs of promoters were predicted using PLACE Plant-CARE^3^ databases.

### Production of the Recombinant ERF Proteins in *E. coli*

The full *VviERFs-c* (GenBank accession no. *VviERF069-c*: KX668860; *VviERF2-c*: KX668861; *VviERF10-c*: KX668862; *VviERF11-c*: KX668863; *VviERF6L7-c*: KX668864) open reading frames, including stop codons, were amplified by RT-PCR using the primers included in Supplementary Table S1. The forward *VviERF069-c, VviERF10-c, VviERF11-c*, and *VviERF6L7-c* primers contained a *Bam*HI site, while the forward *VviERF2-c* primer contained a *Xho*I site. The reverse *VviERF069-c* primer contained a *Hin*dIII site while the reverse *VviERF2-c, VviERF10-c, VviERF11-c*, and *VviERF6L7-c* primers contained an *Eco*RI site. The resulting fragments digested with their respective restriction enzymes were cloned as N-terminal fusion with an amino acid His tag in pTrcHisA vector (Invitrogen, Carlsbad, USA), previously digested with the same enzymes, and transformed into BL21-CodonPlus (DE3)-RIL competent cells. A single recombinant colony of each sequence was grown overnight at 37°C in 5 ml of lysogeny broth (LB) with 50 μg ml^-1^ ampicillin and 35 μg ml^-1^ chloramphenicol with shaking (200 rpm). Then, the overnight cultures were transferred into 500 ml on LB-medium and grown with vigorous shaking (200 rpm) at 37°C until reached an OD_600_ = 0.6. The production of native VviERFs-c was induced by the addition of 1 mM isopropyl-β-D-thiogalactopyranoside. After 4 h of induction at 37°C, bacteria were harvested by centrifugation at 12,000 × *g* for 5 min at 4°C. Pellets were resuspended in 4 ml lysis buffer (50 mM NaH_2_PO_4_, 500 mM NaCl, and 10 mM imidazole, pH 8.0) per gram of bacteria wet weight and 1 mg ml^-1^ of lysozyme. The samples were incubated 30 min on ice followed by three rounds of freezing in liquid nitrogen and thaw at 42°C. When lysates were very viscous RNase A (10 μg ml^-1^) and DNase I (5 μg ml^-1^) were added and incubated 15 min on ice. Afterward, the cell extracts were centrifuged at 10,000 × *g* for 30 min at 4°C. The soluble recombinant proteins were affinity-purified with Ni–NTA agarose resin (QIAexpress, Qiagen, Germany) accordingly to manufacturer instructions for native conditions. The purified fusion proteins were concentrated with buffer exchange into 20 mM potassium phosphate buffer pH 7.0 by ultrafiltration on amicon ultra 3 K (Merck Millipore, Germany). The concentration of recombinant proteins was determined by the Bradford method (Bradford, 1976).

### Gel Electrophoresis and Western Blot Analysis

Protein analyses were performed on 12.5 % sodium dodecyl sulfate polyacrylamide gel electrophoresis using Mini-Protean II Cell (Bio-Rad) equipment as described previously (Romero et al., 2008). Western blots were probed with a mouse anti-6×His monoclonal antibody (1:1000) and a horseradish peroxidase-conjugated antimouse IgG secondary antibody (1:2000) (R&D Systems, Inc.). The immuno-complexes were visualized using the enhanced chemiluminescence detection system (Amersham, GE Healthcare, UK).

### Electrophoretic Mobility Gel Shift Assay

Purified VviERFs recombinant proteins were used to determine DNA binding by electrophoretic mobility gel shift assay (EMSA). Oligonucleotides containing the GCC boxes presented in the promoter regions of different PR genes were synthetized and used as probes, which were biotin-labeled using the Biotin 3′ End DNA Labeling Kit (Thermo Scientific Pierce). Binding of the GCC boxes with the recombinant VviERFs-c proteins was carried out in a 20 μl binding reaction containing 2 μg of recombinant proteins, 50 μM unlabeled DNA, 80 nM biotin-labeled probe, 50 ng μl^-1^ Poly (dI⋅dC), 2.5% (v/v) glycerol, 1× binding buffer. Binding reactions were incubated at room temperature for 20 min. Then reactions were stopped adding 5 μl of 5× loading buffer to each binding reaction and mixed by pipetting up and down. For the competition assays, 500 times of unlabeled probes were added 10 min before the labeled ones. The binding reactions were separated on a 6% native polyacrylamide gel in 0.5× tris-borate-EDTA (TBE) buffer for 45 min to 1 h at 100 V. The gel was transferred to positively charged nylon membrane at 4°C for 1 h at 100 V. When transfer was completed, membranes were crosslinked using a commercial UV-light crosslinking instrument equipped with 254 nm bulbs at 120 mJ/cm^2^. The detection of biotin-labeled DNA was performed by chemiluminescence following Fisher Scientific Pierce manufacturer’s indications. Membranes placed in a film cassette were exposed to X-ray films.

### Statistical Analyses

Data were subjected to one-way analysis of variance using the Fisher’s least significant difference (LSD) test to determinate the level of significance at *P* ≤ 0.05 (Statgraphics Centurion XVII, STSC, Rockville, MD, USA). The relationship between *ERFs* and *PR* gene expression was described as Pearson product moment correlation coefficient (*r*), *P* < 0.05, using expression values normalized to *Actin1* reference gene and afterward to time 0.

## Results

### Isolation and Sequence Analysis of *VviERFs-c* Genes

Five ERF full-length cDNAs were isolated from Cardinal berries and designated as *VviERFs-c* according to the nomenclature system developed by the International Grape Genome Program (IGGP) Super Nomenclature Committee (Grimplet et al., 2014) and following the gene name proposed by Cramer et al. (2014) (**Table 1**, Supplementary Figure S1). The VviERF069-c consisted of 285 amino acids and shared 99.6% identity with VIT_206s0004g08190, the difference being a variation of a single amino acid. VviERF2-c (with 282 amino acids) shared 98.9% identity with VIT_202s0234g00130 and the differences were three variations of single amino acids. VviERF10-c consisted of 204 amino acids, and presented 100% identity with VIT_212s0059g01460. VviERF11-c (220 amino acids) shared 99.1% identity with VIT_219s0014g02240, showing variations of two single amino acids. The analysis of VviERF10-c and VviERF11-c sequences denoted that both contained a conserved EAR repressor motif (ERF-associated amphiphilic repression; DLNXXP) at the C-terminal. VviERF6L7-c (with 277 amino acids) shared 98.2% identity with VIT_216s0013g01050, showing variations of five single amino acids. When examining the theoretical p*I* and molecular mass of the five VviERFs-c, it is noticeable that VviERF069-c, VviERF2-c, and VviERF6L7-c presented the highest molecular weights (above 30 kDa) and although VviERF069-c and VviERF2-c showed a theoretical p*I* of 6.04 and 6.92, respectively, in the case of VviERF6L7 it was 5.70. However, the theoretical molecular mass of VviERF10-c and VviERF11-c was about 23 kDa and both presented a basic p*I* of 8.41 and 8.89, respectively.

**Table 1:** Correspondence between the nomenclature used in this study of table grapes ERFs and previous nomenclatures.

Gene name	Gene name (Licausi et al., 2010a)	Gene name (Cramer et al., 2014)	GrapegenDB 12Xv2 unique ID
*VviERF069-c*	*VvERF055*	*VviERF069*	*VIT_206s0004g08190*
*VviERF2-c*	*VvERF076*	*VviERF2*	*VIT_202s0234g00130*
*VviERF10-c*	*VvERF066*	*VviERF10*	*VIT_212s0059g01460*
*VviERF11-c*	*VvERF063*	*VviERF11*	*VIT_219s0014g02240*
*VviERF6L7-c*	*VvERF104*	*VviERF6L7*	*VIT_216s0013g01050*

**FIGURE 1:**
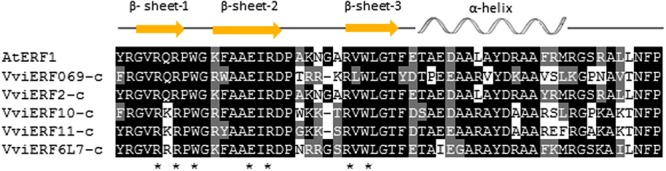
**Alignment of the AP2/ERF domains from the five VviERFs-c and ERF1 from *Arabidopsis thaliana*.** Residues which are identical or similar to the consensus are shaded in black or gray, respectively. Dashes indicate gaps introduced to maximize the alignment. Asterisks below the alignment represent residues that directly make contact with DNA. The three β-sheets and the α-helix are indicated above the figure.

The five VviERFs-c proteins contained an AP2/ERF domain and the alignment with AtERF1 from *Arabidopsis thaliana* showed an identity within this domain that totaled 58.6, 98.6, 69, 69.3, and 79.3% for VviERFs-c, respectively (**Figure 1**). Amino acids, within the β-sheets involved in protein/DNA interactions, are either conserved or replaced by similar residues, with the exception of residue 6 within the first β-sheet. In this case, this residue varies from an uncharged amino acid like glutamine (Q) in VviERF069-c and VviERF2-c, to highly charged residues such as lysine (K) in VviERF10-c and VviERF11-c, or arginine (R) in VviERF6L7-c.

In order to study the phylogenetic relationship between the 122 ERFs from *Vitis* identified by Licausi et al. (2010a) and the five VviERFs-c isolated in the present work, sequences were aligned and a phylogenetic tree was generated (Supplementary Figure S2). Phylogenetic analysis indicated that VviERFs-c could be assigned to previously described ERF classes (Nakano et al., 2006). Thus, 10 groups were drawn and VviERF069-c was located in the group VI, VviERF2-c and VviERF6L7-c were located in the group IX and VviERF10-c and VviERF11-c in the group VIII.

### Response of *VviERFs-c* Genes in Different Tissues of Table Grape Bunches Treated with High CO_2_ Levels during 3 Days at Low Temperature

**FIGURE 2:**
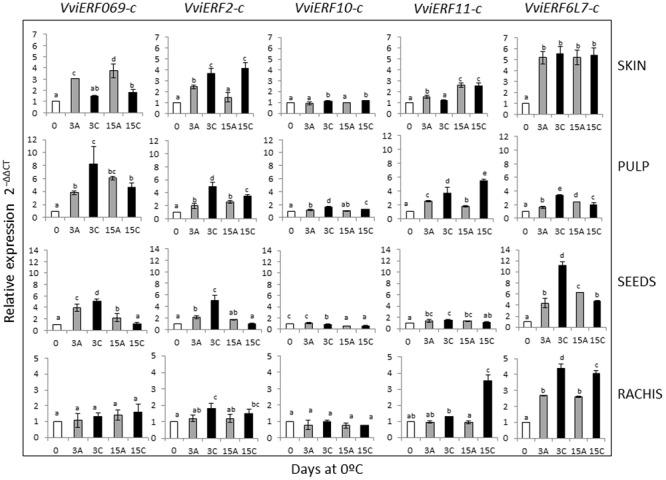
**Effect of a 3-day high CO_2_ treatment and low temperature storage on *VviERFs-c* gene expression in skin, pulp, seeds, and rachis of *V. vinifera* cv.** Cardinal bunches stored at 0°C. Transcript levels of each gene were assessed by RT-qPCR and normalized using actin (*ACT1*) as a reference gene. Results were calculated relative to a calibrator sample (time 0) using the formula 2^-ΔΔCt^. Values are the mean ± SD, *n* = 6. Different letters on bars indicate that the means are statistically different using the LSD test (*P* < 0.05). In the figure, 3A and 15A stand for bunches stored in air (non-treated), 3C stands for bunches treated 3 days with high CO_2_ levels, and 15C stands for grapes stored 12 days in air after the 3-day CO_2_ treatment.

To analyze the mechanisms associated with the beneficial effect of the application of a 3-day gaseous treatment in table grapes stored at 0°C (Sanchez-Ballesta et al., 2006; Rosales et al., 2016), we investigated changes in the five *VviERFs-c* isolated in different fruit and non-fruit tissues such as skin, pulp, seeds, and rachis using the RT-qPCR method with specific primers (**Figure 2**). Our results showed that in most of the tissues analyzed the *VviERFs-c* gene expression was induced after storage under normal atmosphere, although applying high levels of CO_2_ at 0°C caused a greater increase in *VviERFs* transcript accumulation. However, the pattern of expression was different depending on both the tissue and the *VviERFs*. Thus, the storage of non-treated table grapes at 0°C induced the expression of *VviERF069-c, VviERF2-c*, and *VviERF11-c* in the skin. The application of high CO_2_ levels at 0°C modulated the *VviERFs-c* gene expression in this tissue, either reducing the induction observed in the skin of non-treated grapes in the case of *VviERF069-c* and *VviERF11-c* or activating the accumulation of *VviERF2-c* and *VviERF10-c.* The accumulation of *VviERF6L7-c* in the skin was induced irrespective of the storage atmosphere.

In the pulp, the expression of the five *VviERFs-c* was induced after 3 days at 0°C and maintained up to 15 days. However, the application of a 3-day pretreatment with high CO_2_ levels at 0°C caused a greater induction of the five *VviERF* transcripts, although it was transitory and was maintained only in the case of *VviERF11-c* when table grapes were transferred to air.

Regarding the seeds, an increase in *VviERF069-c, VviERF2-c, VviERF11-c*, and *VviERF6L7-c* gene expression during the storage of table grapes under normal atmosphere was also observed, and the application of 3-day high CO_2_ levels resulted in a greater accumulation of these transcripts. By contrast, *VviERF10-c* gene expression decreased in CO_2_-treated samples in comparison to non-treated ones.

In the rachis, *VviERF069-c* and *VviERF10-c* transcript accumulation did not vary either by the storage at low temperature in air or by the application of high CO_2_ levels. By contrast, *VviERF2-c* and *VviERF11-c* gene expression only increased in CO_2_-treated samples, although the accumulation of *VviERF2-c* was observed at the end of the gaseous treatment and in the case of *VviERF11-c* when treated bunches were transferred to air. Finally, *VviERF6L7-c* gene accumulation increased both in treated and non-treated samples, although the increase was higher when CO_2_ was applied.

### *Cis*-Regulatory Elements in the Promoter Regions of *Vcchit1b* and *Vcgns1*

**FIGURE 3:**
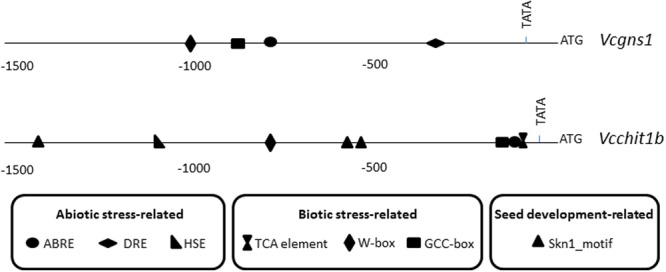
**Location of putative *cis*-acting regulatory elements involved in biotic and abiotic stress and skin development in promoter regions of *Vcgns1* and *Vcchit1b* genes**.

The promoter regions of *Vcchit1b* and *Vcgns1* (including around 1600- or 1100-bp of sequences upstream of the translational start codon, respectively) were obtained using the Genome Walking Kit (Clontech) (**Figure 3**) and is likely to harbor most regulatory elements necessary for driving the regulated transcription of these genes. The sequence identity between the cloned promoters from Cardinal and the corresponding sequences in the PN40024 grapevine reference genome was analyzed. Results indicated that glucanase promoters shared 97.5% identity and chitinase promoters shared 99.7% identity. *In silico* analysis of the promoters performed by PlantCare software identified different *cis*-regulatory elements that were classified into three groups according to their potential responsive functions: abiotic stress-related elements, biotic stress-related elements, and seed development-related elements. The abiotic stress-related elements comprised, among others, ABA-responsive elements (ABRE), DRE, and heat shock-responsive elements (HSE). Within the biotic stress-related elements there were included ethylene-responsive elements (GCC box), salicylic acid-responsive elements (TCA), as well as elicitor-responsive elements (W-box). Seed development-related elements comprised only endosperm-specific expression elements (Skn-1 motif). *Vcgns1* promoter harbored four *cis*-regulatory elements, two related with abiotic and two related with biotic stresses. By other hand, the *Vcchit1b* promoter presented nine *cis*-regulatory elements: three related with seed development, one HSE and one TCA-element. All these mentioned elements were not presented in *Vcgns1*. Both PR promoters had in common the presence of a single W-box, an ABRE motif and a GCC box.

### *Vcchit1b* and *Vcgns1* Gene Expression

**FIGURE 4:**
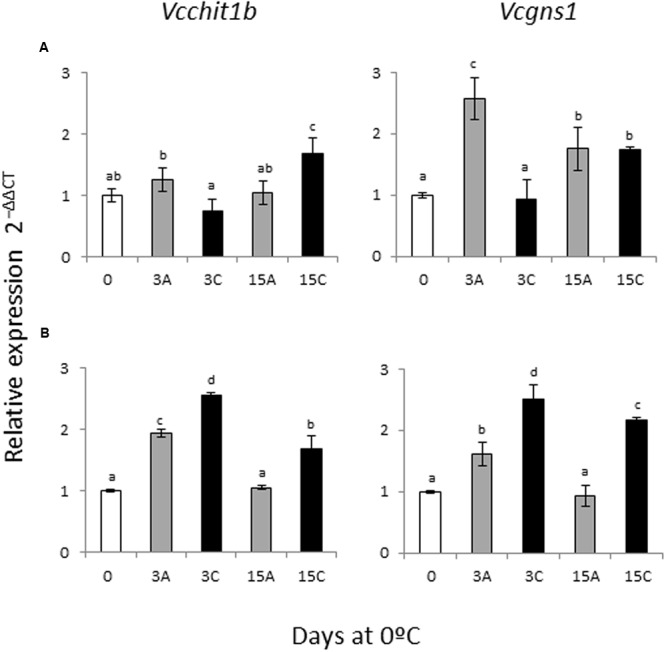
**Effect of a 3-day high CO_2_ treatment and low temperature storage on *Vcgns1* and *Vcchit1b* gene expression in pulp (A)** and rachis **(B)** of *V. vinifera* cv. Cardinal bunches stored at 0°C. Transcript levels of each gene were assessed by RT-qPCR and normalized using actin (*ACT1*) as reference gene. Results were calculated relative to a calibrator sample (time 0) using the formula 2^-ΔΔCt^. Values are the mean ± SD, *n* = 6. Different letters on bars indicate that the means are statistically different using the LSD test (*P* < 0.05). In the figure, 3A and 15A stand for bunches stored in air (non-treated), 3C stands for bunches treated 3 days with high CO_2_ levels, and 15C stands for grapes stored 12 days in air after the 3-day CO_2_ treatment.

To analyze the putative role of *VviERFs-c* modulating PRs, gene expression of *Vcgns1* and *Vcchit1b* was studied in the pulp and rachis of CO_2_-treated and untreated samples by RT-qPCR (**Figure 4**). In the skin, *Vcgns1* and *Vcchit1b* gene expression were not analyzed because they were previously determined by northern blot (Romero et al., 2008; Fernandez-Caballero et al., 2009). *Vcgns1* was significantly induced after 3 days of storage at 0°C in the pulp. Whereas the application of the gaseous treatment did not affect *Vcgns1* gene expression in the pulp, when treated table grapes were transferred to air the accumulation of *Vcgns1* reached values similar to those observed in non-treated samples. However, accumulation of *Vcchit1b* transcripts only increased in the pulp of 3-day CO_2_-treated fruit after transferring to air for up to 12 days (**Figure 4A**). By contrast, both *Vcgns1* and *Vcchit1b* followed the same pattern of expression in the rachis (**Figure 4B**). Thus, both genes were significantly modulated by storage under normal atmosphere after 3 days, decreasing thereafter. The application of a 3-day gaseous treatment caused a greater accumulation of both transcripts in comparison to non-treated samples, which was maintained when treated bunches were transferred to air.

Studying the correlation between *VviERFs-c* and *Vcgns1/Vcchit1b* gene expression (**Table 2**), we observed a significant positive correlation (*r* = 0.575, *P* < 0.05) between the expression of *VviERF11-c* and *Vcchit1b* in the pulp. On the other hand, in the rachis, significant positive correlations were observed between *VviERF2-c* and *Vcgns1/Vcchit1b* gene accumulation (*r* = 0.843 and *r* = 0.756, respectively, *P* < 0.01). Likewise, in the rachis *VviERF6L7-c* and *Vcgns1/Vcchit1b* transcript accumulation showed similar patterns (*r* = 0.877 and *r* = 0.773, respectively, *P* < 0.01). In the pulp, negative correlations were observed in the gene expression of *VviERFs-c* and the two *PR* genes with the exception of *VviERF11-c*. In the rachis, however, the gene accumulation of the five *VviERFs-c* presented positive correlations with *Vcgns1* and *Vcchit1b.*

**Table 2:** Pearson correlations between the expression of *Vcgns1, Vcchit1b* and the expression of *VviERFs* genes in the pulp and rachis of table grapes cv. Cardinal CO_2_-treated and non-treated stored at 0°C for up to 15 days.

	Pulp		Rachis	
	*Vcgns1*	*Vcchit1b*	*Vcgns1*	*Vcchit1b*
*VviERF069-c*	-0.004	-0.128	0.363	0.171
*VviERF2-c*	-0.169	-0.017	0.843^∗∗^	0.756^∗∗^
*VviERF10-c*	-0.186	-0.022	0.219	0.146
*VviERF11-c*	0.119	0.575^∗^	0.512	0.148
*VviERF6L7-c*	-0.223	-0.29	0.877^∗∗^	0.773^∗∗^

*^∗^Correlation is significant at the 0.05 level. ^∗∗^Correlation is significant at the 0.01 level.*

### VviERFs-c are GCC Box-Binding Proteins

**FIGURE 5:**
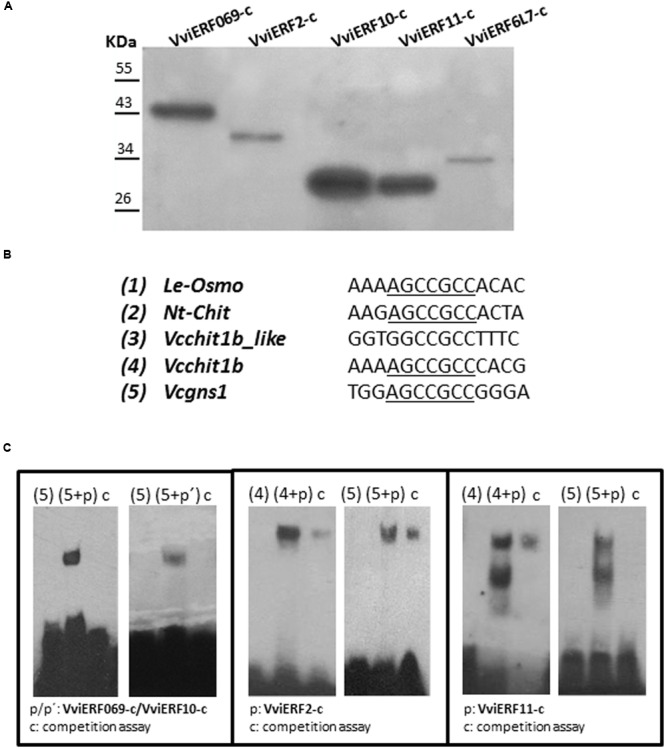
**(A)** Western blots of recombinant VviERFs-c proteins. **(B)** *Cis*-elements used in the EMSA. Le-osmo (AF093743); Nt-chit (X16938). GCC-cores are underlined. **(C)** EMSA analyses were performed with recombinant VviERFs-c proteins. Probes were labeled in the 3′ end with biotin. Five hundred times of unlabeled probe was added prior to the labeled probe for the competition assay.

DNA-binding experiments were carried out (**Figure 5**) with the aim of assessing the binding capacity of the five VviERFs-c to bind the GCC box. The purities of the recombinant VviERFs-c are shown in **Figure 5A**. Several GCC box-containing sequences from tomato (*Le-osmo*), tobacco (*Nt-chit*), and grapes (*Vcchit1b* and *Vcgns1*) were used for mobility gel shift assays (**Figure 5B**). All these probes contained the GCC-core (AGCCGCC), being the differences among them the nucleotides of their flanking regions. Likewise, we have used a probe named *Vcchit1b_like* presented in the promoter of *Vcchit1b* that contains GCCGCC proceeded by G instead of A. None of the five VviERFs-c were able to bind specifically to tomato osmotin and tobacco chitinase GCC boxes (data not shown). We also observed that the recombinant VviERF6L7-c protein was the only one that did not show specific binding to the Vcgns1 GCC box, among the proteins studied (**Figure 5C**). By other hand, binding activity was dramatically reduced or abolished in all cases by competition with high amounts of unlabeled probe. Furthermore, VviERF2-c and VviERF11-c were able to specifically bind to the GCC box from the *Vcchit1b* chitinase promoter. However, it is important to note that no binding activity was observed with the probe *Vcchit1b_like* (data not shown).

## Discussion

The AP2/ERF protein family contains transcription factors that play a crucial role in plant growth and development as well as in various responses to environmental stimuli. Within this transcription factor family, ERFs comprise one of the largest in plants, with 122 and 139 members in *Arabidopsis* and rice, respectively (Nakano et al., 2006). In *V. vinifera*, Zhuang et al. (2009) identified 73 ERF transcription factor genes and thereafter, Licausi et al. (2010a) re-screened the grapevine 8× reference genome identifying 122 ERF members. Although previous studies identified ERFs as being associated with ripening in grapes (Licausi et al., 2010a; Carbonell-Bejerano et al., 2013), reports on the role ERFs play in grape postharvest responses remain poorly documented. Likewise, there have been very few studies in which their putative function in this fruit has been characterized. In a transcriptomic analysis performed previously, we observed that the application of high CO_2_ levels at low temperature activated specific transcriptional modifications depending on the date of harvest, inducing the accumulation of different transcription factors, such as ERFs, in the skin of table grapes harvested at an immature non-commercial stage (Rosales et al., 2016). To further study the role of this transcription factor family, we isolated five VviERFs-c in table grapes harvested at an early commercial stage, and characterized their expression pattern in fruit and non-fruit tissues during postharvest storage by combining both low temperature and high CO_2_ levels. It is important to note that three of the *VviERFs-c* isolated (*VviERF069-c, VviERF2-c*, and *VviERF6L7-c*) were identified in the transcriptional analysis mentioned above as genes up-regulated by application of gaseous treatment (Rosales et al., 2016).

ERF-related transcription factors are assumed to be involved in biotic and abiotic stress responses in plants (Lorenzo et al., 2003; Chen et al., 2008; Licausi et al., 2010b; Pan et al., 2012; Zhao et al., 2012; Yang et al., 2016). Storage of table grapes at 0°C is recommended to prolong their postharvest quality. Although grapes are classified as a chilling tolerant fruit, a significant increase in ion leakage (which was used as an indicator of membrane damage during chilling and senescence) was observed in non-treated fruit in comparison to CO_2_-treated ones (Rosales et al., 2016). To date, there has been no comprehensive analysis regarding the transcriptional responses of *ERF* genes to postharvest storage conditions associated with mechanisms underlying abiotic stress responses, such as low temperature or modified atmosphere. In this work, we observed that although the expression of *VviERFs-c* was induced by low temperature storage under normal atmosphere in most of the tissues analyzed, the 3-day high CO_2_ treatment at 0°C activated a greater induction. The expression of *VviERF069-c, VviERF10-c, VviERF11-c*, and *VviERF6L7-c* was tissue dependent; however, in the case of *VviERF2-c*, the pattern of accumulation was similar in all the tissues analyzed showing an induction in non-treated samples and a greater accumulation at the end of the gaseous treatment. It is interesting to note that the effect of the high CO_2_ level in the modulation of the expression of different *ERFs* was more evident in the pulp and seeds among the samples analyzed. The results, obtained in this work, are in concordance with a previous work where we observed that applying high CO_2_ levels at 0°C for 3 days induced the expression of *CBF1* and *CBF4*, belonging to the DREB subfamily (Fernandez-Caballero et al., 2012), indicating that the AP2/ERF family seems to play a prominent role in the beneficial effect of the gaseous treatment to overcome low temperature storage in table grapes. Likewise, the fact that several *ERFs* were significantly up-regulated in high temperature ripening grapes suggests that this transcription factor family could trigger the maintenance of thermotolerance response in grapevine berries (Carbonell-Bejerano et al., 2013). Another interesting result from our expression profile analyses is the fact that, in most cases, *VviERFs-c* transcript accumulation was induced in non-treated bunches after 3 days at 0°C and the levels were maintained or even increased at day 15. By contrast, in CO_2_-treated bunches most of the *VviERFs-c* showed the “switch mode” expression pattern, presenting an induction at the end of the gaseous treatment followed by a rapid decline. It is important to note that similar results were observed in kiwifruit (Yin et al., 2012) and papaya (Li et al., 2013) but as a response to low temperature postharvest storage. These authors suggested that this mode of modulation of *ERFs* is a fruit response to low temperature stress and is a factor in turning on *ERFs* and thus activating or repressing gene expression, which might be critical for fruit protection from stress damage during postharvest, and in our case could play an important role on the beneficial effect of the 3-day gaseous treatment at 0°C on table grapes. On the other hand, whereas the accumulation of *ERF* transcripts by low temperature has been previously described, the effect of high CO_2_ levels on *ERF* expression has been analyzed only in kiwifruit (Yin et al., 2012), where the application of 5% of CO_2_ at 20°C for up to 7 days activated the accumulation of several ERFs, and in persimmon (Min et al., 2014), where exposition to 95% of CO_2_ during 1 day at 20°C, to remove astringency, induced the accumulation of most of the *ERFs* analyzed. To our knowledge, however, this is the first time that an induction of *ERFs* gene expression by the combination of high CO_2_ levels and low temperature has been described in different reproductive organs and fruit tissues.

By analyzing phylogenetic results together with those of *ERFs* gene expression, we observed that genes belonging to the group IX (*VviERF2-c* and *VviERF6L7-c*), according to the ERF groups designated by Licausi et al. (2010a), showed a greater accumulation of their transcripts by the effect of high CO_2_ levels in all the samples analyzed, except for the skin in the case of VviERF6L7-c. However, VviERF10-c and VviERF11-c belong to group VIII and, in general, their transcript levels were not induced by the gaseous treatment, or did it slightly. Both, VviERF10-c and VviERF11-c, contained an EAR repressor domain at the C-terminal and it has been reported that the repressor-type ERF proteins could play key roles in several biological functions by negatively regulating genes involved in developmental, hormonal, and stress signaling pathways (Ohta et al., 2001).

In a recent work, Sun et al. (2016) indicated that the enhanced expression of *VaERF057* under cold stress in grapevine was possibly directly induced by ethylene, the release of which was promoted by low temperature. Likewise, their results of the evolution of both ACC (1-aminocyclopropane-1-carboxylic acid) content and ACO (ACC oxidase) activity strongly support the increase in ethylene production in grapevines during the early stage of cold stress. Results obtained by Muñoz-Robredo et al. (2013), confirmed that ethylene may have a role in some aspects of the grape ripening process. They also highlighted the potential use of *VvACO1*, as a molecular marker for identifying grape veraison stages. In a previous work, we observed that the development of rachis browning in non-treated table grapes was linked to an induction of *ACS1* and *ACO1* gene expression. By contrast, *ACO1* and *ACS1* transcript levels were markedly induced in grape skin treated with high CO_2_ levels, while in non-treated skin the induction was lower or appeared later during storage (Rosales et al., 2013). We analyzed the correlations between the expression of *ACO1, ACS1* and the five *VviERFs-c* in fruit and non-fruit tissues of treated and non-treated table grapes stored at 0°C and did not find any significant result (data not shown), not being able to establish a relationship between the expression of ethylene biosynthetic genes and *VviERFs-c*.

As transcription factors, the ERFs play important roles in stress response by regulating the expression of downstream stress-related genes, interacting with the GCC box present in their promoters (Ohme-Takagi and Shinshi, 1995; Yamaguchi-Shinozaki and Shinozaki, 2006; Pirrello et al., 2012; Dong et al., 2015). Our expression analyses of two PR genes, *Vcchit1b* and *Vcgns1*, from Cardinal table grapes, denoted significant correlations with *VviERF2-c* and *VviERF6L7-c* gene expression in rachis, and between *VviERF11-c* and *Vcchit1b* in pulp. In this study, we also provided evidences that recombinant VviERF2-c and VviERF11-c bound specifically to the GCC box presented in the promoter of the above mentioned PR genes. However, VviERF069-c and VviERF10-c can only bind to the GCC box of the *Vcgns1* promoter and VviERF6L7-c showed no affinity for any of the GCC box studied. Previous works stressed the putative role of the GCC box flanking regions in impacting the binding activity of the ERFs (Tournier et al., 2003; Pirrello et al., 2012). Specifically, results obtained by Pirrello et al. (2012) suggested that the two nucleotides directly flanking the GCC box at position N_4_ and N_11_ are essential for the binding of ERFs to the GCC box, determining their nature whether or not the *cis*-element is functional. In this sense, we observed that the change of A into G at position 4, avoided the affinity to the *Vcchit1b* box of all the VviERFs-c. Mutation of A_11_ to any of the three other nucleotides resulted in a dramatic loss of affinity to the GCC box (Pirrello et al., 2012). Curiously, although we have not used mutated versions of the GCC box of the *Vcchit1b* and *Vcgns1* promoters, the presence of a C or G at position 11 did not avoid the affinity of the VviERFs-c studied. However, when we used the GCC box presented in tomato osmotin and tobacco chitinase promoters with an A at position 11, no binding with the five VviERFs-c was observed. By other hand, Tournier et al. (2003) also suggested that variation in amino acid composition within the binding domain may also affect the binding affinity of ERFs to the GCC box. Changing residue 6 from basic charged, lysine (K) or arginine (R), to uncharged, glutamine (Q), did not alter the specificity of interaction with the *cis*-element, but greatly decreased binding affinity. However, although further analyses would be needed, it is important to note that we did not detect binding affinity with VviERF6L7-c which it was the only VviERF tested in this work that presented an arginine. In conclusion, we speculate that *ERF* transcription factor family might play a role in the beneficial effect of the gaseous treatment on table grape quality based on the results of RT-qPCR together with the EMSA analysis that denote the putative regulation of the expression of two PR genes interacting with the GCC box presented in their promoters. In this sense, the fact that *VviERF2-c* gene expression showed, on one hand, an induction by high CO_2_ levels in all the tissues analyzed and, on the other hand, a significant correlation with the expression of the two PR genes analyzed, together with the *in vitro* binding affinity observed with the GCC box of their promoters, make it a good candidate for further analysis in order to improve table grape quality by breeding.

## Author Contributions

IR contributed to ERFs isolation, RNA extraction, RT-qPCR analysis, EMSA analysis, statistical analyses, edited and collaborated in the first draft of the manuscript. MV-H contributed to PR gene expression. ME and CM improved the manuscript. MS-B designed the research and prepared the first draft of the manuscript. All authors have read and approved this manuscript.

## Conflict of Interest Statement

The authors declare that the research was conducted in the absence of any commercial or financial relationships that could be construed as a potential conflict of interest.

## Footnotes

1 http://genomes.cribi.unipd.it/grape/

2 http://www.ch.embnet.org/software/LALIGN_form.html

3 http://bioinformatics.psb.ugent.be/webtools/plantcare/html/
