# Supplementary material for: Expression Profiles and DNA-Binding Affinity of Five ERF Genes in Bunches of Vitis vinifera cv. Cardinal Treated with High Levels of CO2 at Low Temperature
Source: Front Plant Sci. 2016 Nov 28;7:1748. doi: 10.3389/fpls.2016.01748 (PMC5124697; doi:10.3389/fpls.2016.01748)
Supplement: Supplementary file 2 [file Image_1.PDF]

Supplementary Figure S1. Multiple amino acid sequence alignment of the five VviERFs from table grapes cv. Cardinal and their homologs according to Licausi et al. (2010a). The dashes indicate gaps introduced for better alignment. Identical amino acids are shaded in black and conserved substitutions are shaded in grey.

```
VviERF069-c    1 MLGCVRKSMGSDFLMPIRPAVKFSEHVVTTSKHMQELESVSASECLSGRRRVRHRVVRII
VvERF055       1 MLGCVRKSMGSDFVMPIRPAVKFSEHVVTTSKHMQELESVSASECLSGRRRVRHRVVRII
VviERF2-c      1 --------MCDYSSNXSSDFALLESVRRHLFDDSDSRRFDAPLYCRSNSFSSLFETGGEL
VvERF076       1 --------MCDYSSNPSSDFALLESVRRHLFDDSDSRRFDAPLYCRSNSFSSLFETGGEL
VviERF10-c     1 ------------------------------------------------------------
VvERF066       1 ------------------------------------------------------------
VviERF11-c     1 ------------------------------------------------------------
VvERF063       1 ------------------------------------------------------------
VviERF6L7-c    1 -MAEEASSLHLIHQQLLFDFESFEGFVSHVNVPSQTSTSDS-----SPSTSDIVPLSNYS
VvERF104       1 -MAEEASSLHLIHQQLLFDFESFESFVSHVNDPSQTSTSDS-----SPSTSDIIPLSDYS

VviERF069-c   61 HTDGDATDSSSDDEVELVQRVKRHVTEISLQPSVESPQKEPTKKRVLR------------
VvERF055      61 HTDGDATDSSSDDEVELVQRVKRHVTEISLQPSVESPQKEPTKKRVLR------------
VviERF2-c     53 PLKEDDSDDMVIYGFLRDAASGGWTPTLAPLFSETASYGFSTAPAVAVKSEPEVFPAEVI
VvERF076      53 PLKEDDSDDMVIYGFLRDAASGGWTPTLAPLFSETASHGFSTAPAVAT------------
VviERF10-c     1 -----------------------------MRRGRATARDAPQQQEVR-------------
VvERF066       1 -----------------------------MRRGRATARDAPQQQEVR-------------
VviERF11-c     1 ----------------------------------MAPRDKPTGVTAG-------------
VvERF063       1 ----------------------------------MAPRDKPTGVTAG-------------
VviERF6L7-c   55 NLHEDENNPFLLHCSTLAPSGFFQFETKSPKNSTLSHRRPPLSISVPQ------------
VvERF104      55 NLHEDENNPFLLHCSTSAPSGFCQFETKSPKYSTLSHRRPPLSISVPQ------------

VviERF069-c  109 --------------LPESESTRRKKFRGVRQRPWGRWAAEIRDPTRR-KRLWLGTYDTPE
VvERF055     109 --------------LPESESTRRKKFRGVRQRPWGRWAAEIRDPTRR-KRLWLGTYDTPE
VviERF2-c    113 GVPEKTMDPPAKLPAPAVVPAKGKHYRGVRQRPWGKFAAEIRDPAKNGARVWLGTFETAE
VvERF076     101 ------VDPPAKLPAPAVVPAKGKHYRGVRQRPWGKFAAEIRDPAKNGARVWLGTFETAE
VviERF10-c    19 -----------------NQG-KDVRFRGVRKRPWGRFAAEIRDPWKK-TRVWLGTFDSAE
VvERF066      19 -----------------NQG-KDVRFRGVRKRPWGRFAAEIRDPWKK-TRVWLGTFDSAE
VviERF11-c    14 -----------------ATGNKEIRYRGVRKRPWGRYAAEIRDPGKK-SRVWLGTFDTAE
VvERF063      14 -----------------ATGNKEIRYRGVRKRPWGRYAAEIRDPGKK-SRVWLGTFDTAE
VviERF6L7-c  103 --------PTVSQSPAESDSGDIRHYRGVRRRPWGKFAAEIRDPNRRGSRVWLGTFETAI
VvERF104     103 --------PTVSQSPAESDSGDIRHYRGVRRRPWGKFAAEIRDPNRRGSRVWLGTFETAI

VviERF069-c  154 EAARVYDKAAVSLKGPNAVTNFPSVVKTESVATAGQSQSETCSLPSVTASSPTSVLRYNE
VvERF055     154 EAARVYDKAAVSLKGPNAVTNFPSVVKTESVATAGQSQSETCSLPSVTASSPTSVLRYNE
VviERF2-c    173 DAALAYDRAAYRMRGSRALLNFPLRVNSGEPDPVRVTSKRSSPEPSSSSTSSSSSDNSSP
VvERF076     155 DAALAYDRAAYRMRGSRALLNFPLRVNSGEPDPVRVTSKRSSPEPSSSSTSSSSSDNSSP
VviERF10-c    60 DAARAYDAAARSLRGPKAKTNFPLSSPAVPLTVPFQQPSQYQIQDQNHSHQHQPQRPTTS
VvERF066      60 DAARAYDAAARSLRGPKAKTNFPLSSPAVP---------QYQIQDQNHSHQHQPQRPTTS
VviERF11-c    56 EAARAYDAAAREFRGAKAKTNFPSPTDLAAAAATTANRSPSQSSTVESSSREALSPGAIA
VvERF063      56 EAARAYDAAAREFRGAKAKTNFPSPTDLAAAAATTANRSPSQSSTVESSSREALSPGAIA
VviERF6L7-c  155 EGARAYDRAAFKMRGSKAILNFPLEADNWSGSDPPATSGRKRVRDSETEERQQVEIKVLK
VvERF104     155 EGARAYDRAAFKMRGSKAILNFPLEADNWSGSDPPATSGRKRGRDSETEERQQVEIKVLK

VviERF069-c  214 EAPFNGFGYCDVDAFG-------------------------FDFDVPLTLPDIFSNKHLE
VvERF055     214 EAPFNGFGYCDVDAFG-------------------------FDFDVPLTLPDIFSNKHLE
VviERF2-c    233 KRRKKVSSLAAPAVAP-------------------------ATAQTGIEIGKSMEGSQAG
VvERF076     215 KRRKKVSSLAAPAVAP-------------------------ATAQTGIEIGKSMEGSQAG
VviERF10-c   120 GMSSTVESFSGPRLSNPPSAP------------------PRPRLRAPPVNDCQSDCD-SS
VvERF066     111 GMSSTVESFSGPRLSNPPSAP------------------PRPRLRAPPVNDCQSDCD-SS
VviERF11-c   116 GPPALDLNLSHPAAAGQFSAVRYPAVGVFPIAQPLFFFEPFSRPEKPKTHRDMFDLDRAV
VvERF063     116 GPPALDLNLSHPAAAGQFSAVRYPAVGVFPIAQPLFFFEPFSRPEKPKTHRDMFDLDRAV
VviERF6L7-c  215 QEEHLPESDCTLAAAS-------------------------NVLGVCPLTPSNWRAVWEE
VvERF104     215 QEEPLPESDCTLAAAS-------------------------NVLGVCPLTPSNWTAVWEE

VviERF069-c  249 EDEFGEFDPDDFLVFETSLPLLHSXKSDEGECRVGGL--------
VvERF055     249 EDEFGEFDPDDFLVFETSLPLLHSNKSDEGECRVGGL--------
VviERF2-c    268 YEVAQLTHGPQLLVR------------------------------
VvERF076     250 YEVAQLTHGPQLLVR------------------------------
VviERF10-c   161 SSVIDECDGDLTSSSRKPLPFDLNLLPPSEDGDDDDLDATALCL-
VvERF066     152 SSVIDECDGDLTSSSRKPLPFDLNLLPPSEDGDDDDLDATALCL-
VviERF11-c   176 ADFHPAIAGSVHSDSDSSSVVDFNYHDRGTRLLDLDLNHPPAEVA
VvERF063     176 ADFHPAIAGSVHSDSDSSSVVDFNYHDRSTRLLNLDLNHPPAEVA
VviERF6L7-c  250 GDMEGMFHLPPLTPLSPHPWIAYSQLIV-----------------
VvERF104     250 GDMEGMFHLPPLTPLSPHPWIAYSQLIV-----------------
```
