# Supplementary material for: Expression Profiles and DNA-Binding Affinity of Five ERF Genes in Bunches of Vitis vinifera cv. Cardinal Treated with High Levels of CO2 at Low Temperature
Source: Front Plant Sci. 2016 Nov 28;7:1748. doi: 10.3389/fpls.2016.01748 (PMC5124697; doi:10.3389/fpls.2016.01748)

Supplementary Material Figure S2. Phylogenetic tree based on the 122 known ERFs sequences from *Vitis vinifera*. VviERFs-c were included and grouped in their respective families. The ten families identified according to Licausi et al. (2010a) are in colour.

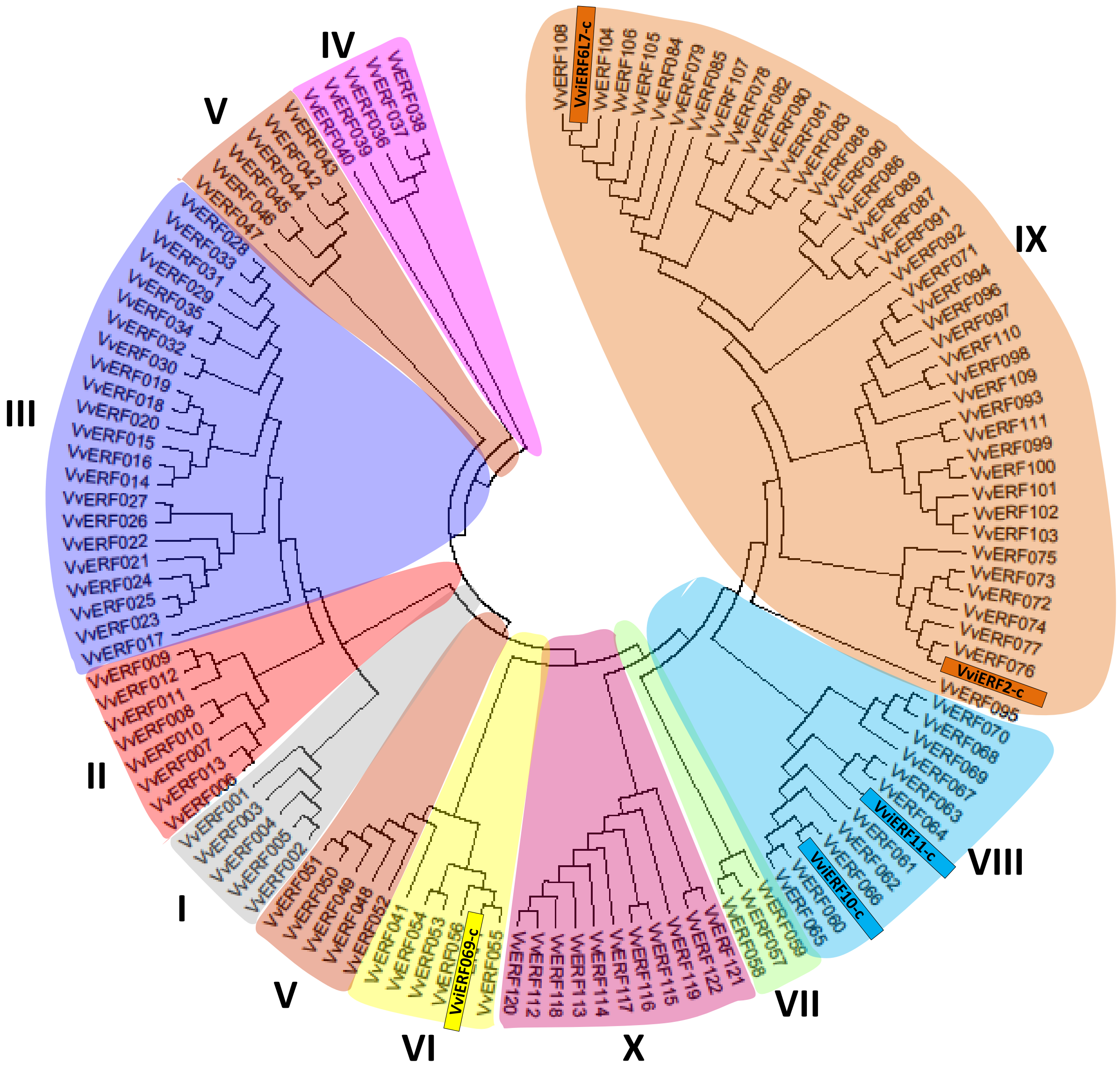

Supplement: Supplementary file 3 [file Image_2.PDF]
